# Hydrazone-Containing Triblock Copolymeric Micelles for pH-Controlled Drug Delivery

**DOI:** 10.3389/fphar.2018.00012

**Published:** 2018-01-23

**Authors:** Peilan Qi, Xiaohe Wu, Lei Liu, Huimin Yu, Shiyong Song

**Affiliations:** Institute of Pharmacy, Pharmaceutical College of Henan University, Kaifeng, China

**Keywords:** triblock copolymer, pH-sensitive, cytotoxicity, micelles, tumor targeting

## Abstract

In this study, the structure–activity relationship of amphiphilic block copolymer micelles as nanosized drug delivery system was revealed. Firstly, a biodegradable triblock polymers PEG-DiHyd-PLA containing hydrazone bond was synthesized through the ring-opening polymerization. In this method, PEG-DiHyd-Phenol was used as the initiator and L-lactide as the monomer. Then, the polymeric micelles were formed and used as nano-drug carriers with pH sensitivity. The structure and composition of the polymer were characterized by infrared (IR), nuclear magnetic resonance (^1^H-NMR), and gel permeation chromatography (GPC), we characterized the self-assembling process of the triblock polymers and the pH sensitivity of the micelles by the means of transmission electron microscopy (TEM), dynamic light scattering method (DLS). Doxorubicin (DOX) acts as the model drug, and we researched the capacities of drug loading and release *in vitro* of the micelles. MTT experiments showed that the blank micelles of PEG-DiHyd-PLA were not cytotoxic to tumor cells (HepG-2, MCF-7) and normal cell (L-02 cells), but the DOX loaded ones displayed more toxicity than the ones without hydrazone, which was consistent to the further confocal laser scanning microscopy and flow cytometry study.

## Introduction

In the past two decades, a large number of nanoparticulate drug delivery systems have been extensively explored as in cancer treatment. Some formulations in the form of liposome, polymer–drug conjugates, and micelle particulate have found their applications in clinics and even more are advanced to the stage of clinical trials. Recently, more sophisticated nano-system have been developed to increase the therapeutic efficacy of cancer by controlling of the drug release temporally or spatially. Stimuli-response functionality is becoming increasingly important due to applications in biotechnology and the crafting of smart materials. Stimulus (Xing et al., 2011), such as pH (Hrubý et al., 2005; Huang et al., 2015; Mei et al., 2016), temperature (Seo et al., 2015), reductant (Deng et al., 2015), and so on (Cheng et al., 2015), were applied to modulate the release profile of the therapeutic agents. Polymeric micelles that possess stimuli-responsive properties have been demonstrated their great potentials in maximizing the therapeutic efficacy by prolonging circulation life of drug, and minimizing side effects (Makino et al., 2015).

It was reported that it is more acidic around tumor than in blood and normal tissue (Webb et al., 2011). The inherent characteristic of tumor tissue makes pH-sensitive drug delivery systems more suitable for cancer chemotherapy. Furthermore, after endocytosis of the pH-sensitive micelles, an accelerated release of the payload occurs in endosomes and lysosomes, which have the low-pH of pH 5.5–6.0 and pH 4.5–5.0, respectively (Zheng et al., 2013).

A pH sensitive drug delivery system can be formed chemically or physically. Polymer-drug conjugate is one of the pH-responsive drug delivery systems that bearing with acid sensitive linkages between therapeutic molecule and polymer. Ulbrich (Hrubý et al., 2005; Ulbrich and Šubr, 2010) and Kataoka (Bae et al., 2003) conjugated block copolymers with doxorubicin (DOX) with an acid labile hydrazone containing linkage. The conjugates can form micelles and a boost release of DOX were found in an acidic environment upon the cleavage of hydrazone bonds. Hu et al. (2010) prepared biodegradable polymeric micelles with DOX conjugated block copolymer via hydrazone and carbamate linkage for DOX and the hydrazone ones have higher pH-sensitivity than the others. While, the frequently used hydrazone bond can only be formed between the DOX and hydrazine motif containing polymer. Anticancer drugs, such as paclitaxel, camptothecin and gemcitabine, are not appropriate for the linkage of hydrazone.

Physical entrapment of hydrophobic anticancer drugs in the core of a pH-responsive polymeric micelles is another way of forming a pH responsive system. In this case, pH sensitive parts are fixed on the body of carrier, on the side chain or backbone of the copolymer which forms the micelle, where sufficient structural changes are initiated in the low pH environment, triggering a boost release of drug simultaneously. Ding et al. (2009) connected poly(ethylene glycol) with stearic acid via a Schiff base bond linkage to form a pH-sensitive amphiphilic molecule mPEG-b-C18. The cleavage of the Schiff base bond under acidic condition resulted in disassociation of micelle and accelerated drug release. It is obvious that this kind of pH sensitive linkage is applicable for DOX but not limited to. The chemistry of pH-sensitive bond determines the performance of the drug delivery systems. There should be a perfect sensitivity that be able to hydrolyze quickly in acidic environments and stay unchanged in others. Acid labile linkages such as hydrazone (Bae et al., 2003; Hrubý et al., 2005; Hu et al., 2010; Ulbrich and Šubr, 2010), acetal (Gillies et al., 2004; Lu et al., 2010), orthoester (Tang et al., 2011; Cheng et al., 2012), citraconic amide (Cao et al., 2014), and Schiff base bonds (Ding et al., 2009) were reported. Among them, hydrazones were studied extensively for the easy preparations, moderate stability and favorable sensitivity.

In addition, the nature of micelle-forming amphiphilic copolymer should be also considered with respect to the biocompatibility, biodegradability, and capacity of drug loading. Usually biodegradable polymers such as polyester, poly (amino acid), and poly (anhydride) are core-forming materials and polyethylene glycols (PEG) (McPherson et al., 1998; Vonarbourg et al., 2006) form hydrophilic shell of micelles. The polyesters are preferred for their good biocompatibility and degradability (Witschi and Doelker, 1998). Poly (lactic acid) is widely used in drug delivery systems for its moderate degradation rate (Sinha et al., 2004).

In our previous studies, hydrazone containing di-block copolymers were used to form pH-sensitive carriers (Qi et al., 2017). Furthermore, with the discovery of more and more biocompatible polymer materials and atom transfer radical polymerization (ATRP) is becoming more and more mature (Cavallaro et al., 2014; Park et al., 2014; Ran et al., 2014; Visnevskij et al., 2014), triblock polymer has become favorable. Triblock polymers also can be used as drug carriers that aremore stable and controllable (Han et al., 2016). In this work, it was started with rational designing of PEG-based marcro-initiators with hydrazone bond imbedded, then hydrophobic polyester segment was incorporated by ring-opening polymerization. Micelles from the amphiphilic block polymer are both biodegradable and pH sensitive, for the controlled release of DOX.

## Materials and methods

### Materials

Polyethylene glycol (PEG; Mn = 6,000) was purchased from Sigma-Aldrich (St. Louis, MO, USA) and dried in a vacuum oven at 70°C before use. L-Lactide (99.5%, Jinan Daigang Biomaterials Co. Ltd., Jinan, China), stannous octanoate [Sn(Oct)_2_, 95%, Sigma-Aldrich, St. Louis, MO, USA], 4-carboxybenzaldehyde (98%, Shanghai Darui, Shanghai, China), N,N′-dicyclohexylcarbodiimide (DCC, 98% Shanghai Darui, Shanghai, China), 4-dimethylaminopyridine (DMAP, 98% Shanghai Darui, Shanghai, China), methyl 4-hydroxybenzoate (98%, Shanghai Darui, Shanghai, China), and hydrazine hydrate aqueous solution (80%,Tianjin Kemiou, Tianjin, China) were used as received. Doxorubicin hydrochloride (Dalan Meilun Biotech., Dalian, China) was stirred with TEA (3 equiv.) in DMSO overnight before the solvent was evaporated using a rotary evaporator to get doxorubicin, i.e., DOX. All organic solvents were analytical reagents and used as received, except that toluene was dried by the sodium method to get anhydrous toluene.

### Preparation of tri-block copolymer PEG-DiHyd-PLA

#### Synthesis of dialdehyde polyethylene glycol (CHO-PEG-CHO)

CHO-PEG-CHO was prepared according to the reported procedure with some modifications (Ding et al., 2009). Firstly, PEG (8 g) in dichloromethane (DCM) (100 mL) reacted with 4-carboxybenzaldehyde in the presence of DCC (3 g) and DMAP (0.5 g) for 72 h in the oil bath of 40°C. Secondly, the solution was filtered and remove the white precipitate impurities of generated. After that, the filtrate was poured into a large amount of ether for precipitation and the solid was recrystallized three times with isopropyl alcohol. Finally, the product is dried in a vacuum drying chamber at 40°C to obtain a white powder solid. CHO-PEG-CHO white powder was obtained with a yield of 92.4%. Nuclear magnetic resonance (^1^H-NMR) (400 MHz, CDCl_3_): δ10.12 (Ar-CHO), δ8.21, δ7.97 (aromatic protons), δ3.65 (–OCH_2_CH_2_O–), δ4.52(–COOCCHO–).

#### Synthesis of (PEG-DiHyd-Phenol) containing a hydrazone bond initiator

Fristly, 4-hydroxybenzoylhydrazine was synthesized according to the previous method (Zheng et al., 2007). Then, 4-Hydroxybenzoichydrazine (0.3 g) reacted with CHO-PEG-CHO (4 g) in 30 ml methanol and 10 ml N,N-methyl formamide (DMF) at 68°C for 18 h. PEG-DiHyd-Phenol was also obtained by precipitation in ethylether, and yellow powder was obtained. ^1^H-NMR (400 MHz, CDCl_3_): δ8.41 (Ar-CH = N), δ10.58 (Ar-OH), δ3.60 (–OCH_2_CH_2_O–).

#### Synthesis of triblock copolymer PEG-DiHyd-PLA by ring-opening polymerization

PEG-DiHyd-Phenol and L-lactide reacted in anhydrous toluene in the presence of stannous octanoate (100 μL). PEG-DiHyd-PLA with different molecular weights were synthesized by varying the ratio between PEG-DiHyd-Phenol and L-lactide. The reactions were conducted at 110°C under the protection of nitrogen gas for 24 h. The copolymer was precipitated three times with cold ethyl ether. It was dried at 45°C in vacuum. PEG-DiHyd-PLA were obtained with a yield of 72%. ^1^H-NMR (400 MHz, CDCl_3_): δ3.64 (–OCH_2_CH_2_O–); δ8.42 (Ar–CH = N); δ5.21 [protons on poly(L-lactic acid) part]; and δ8.09, δ7.92, δ7.84, and δ6.98 (aromatic protons). Meanwhile, PLA-PEG-PLA copolymers without hydrazone linkage were also prepared as pH non-responsive counterpart using PEG as the initiator.

### Characterization

An AVATAR360 (Nicolet, USA) and an AVANCE 400 spectrometer (Brucker, Switzerland) were used to determine the chemical structure of the polymers. A Damn Eos (Wyatt, USA) gel permeation chromatograph (GPC) instrument equipped with Phenogel 10E6A column and an OPTILAB rEX refractive-index detector was used to determine the molecular weight and polydispersity. Tetrahydrofuran (THF) was used as the eluent at a flow rate of 1.0 mL/min at 30°C and polystyrene standards for the calibration. A Zetasizer Nano-ZS90 (Malvern Instruments, UK) and Transmission electron microscopy (JEM-100CX II TEM) were employed to determine the size and the morphology of the micelles.

### Formation of the pH-sensitive micelles

PLA-PEG-PLA or PEG-DiHyd-PLA (30 mg) dissolved in THF 2 mL was added into 40 mL pure water by dropwise. The mixture was stirred for 36 h at room temperature. The sizes evolution in different solutions of the micelles were measured to determine the pH sensitivity. Ten milliliters freshly prepared micelle dispersions was adjusted to pH 5.0 or pH 4.0. The sizes were measured on DLS after 24 h incubation at 37°C with shaking. CMC was determined using pyrene as a fluorescence probe (Xu et al., 2016).

### Drug loading and release

DOX was loaded into the micelles by solvent evaporation method. Typically, copolymers (25 mg) and DOX (2 mg) were dissolved in 1 mL acetone. The solution was added dropwise into 30 mL pure water stirring. Then, the micellar solution was dialyzed against water for 36 h. After filtered through a 0.22 μm syringe filter to remove undissolved DOX, DOX-loaded micelle dispersion was freeze-dried. The drug loading content (DLC was determined by the measurement of fluorescence of DOX (excitation wavelength at 481 nm and emission wavelength at 558 nm). DLC was calculated by the formula below:

$$\begin{array}{l} \text{DLC }(\text{wt }\%)=(\text{weight of loaded drug/weight of drug loaded}\\ \text{ micelles})\times 100\% \end{array}$$

The *in vitro* release experiments of DOX were conducted at 37°C. Dialysis bag (molecular weight cut-off: 8,000–14,000) filled with 3 mL micellar solution was sealed and immersed in 40 mL buffers solution. Three buffer solutions were used: acetate buffer (0.01 M, pH = 4), acetate buffer (0.01 M, pH = 5), and PBS (0.01 M, pH = 7.4). At desired time intervals, 4 mL of solution outside was taken out for fluorescence measurement. Meanwhile, 4 mL fresh medium was replenished. Cumulative released DOX was calculated according to following formula:

$$\begin{array}{l} E_{r}=\frac{V_{e}\overset{n-1}{\underset{1}{\sum }}C_{i}+V_{0}C_{n}}{m_{drug}} \end{array}$$

In this equation, *E*_*r*_ means cumulative release of DOX (%); *V*_*e*_ means volume to be taken very time (mL); *V*_0_, the volume of medium (mL); *C*_*i*_, concentration when certain volume to be taken (μg/mL); *m*_*drug*_, total mass of DOX contained in the release system (μg); *n*, sampling times.

#### *In vitro* toxicity evaluation

MTT assay was applied to evaluate the cytotoxicity of the blank micelles, DOX-loaded micelles, using HepG-2, MCF-7, and normal L-02 cells (from the Shanghai cell bank of the Chinese Academy of Sciences, Shanghai, China). Cells were seeded and incubated for 24 h (37°C, 5% CO_2_) on a 96-well plate. The cell density is 5 × 10^3^ cells per well in 100 μL of 1640 medium (containing 10% FBS). 24 h later, the medium in each well was removed and 100 μL DOX-loaded micelles or free DOX solution were added into the wells. Each concentration has four replicates. Each sample was performed in quintuplicate. After incubation for 48 h, and the viability of cells was measured using the methylthiazoletetrazolium method. Cell viability (%) was calculated by the following equation (Ahmad et al., 2014):

$$\begin{array}{l} \text{Cell Viability }\left(\text{\%}\right)=\left({\text{A}}_{\text{sample}}/{\text{A}}_{\text{control}}\right)\times 100 \end{array}$$

where A_sample_ and A_control_ is absorbance of the sample well and control well, respectively. Data are presented as average SD ± (*n* = 3).

### Confocal laser scanning microscopy

HepG-2 cells were seeded on the cover slips in culture dish with a density of 8 × 10^4^ cell using RPMI-1640 medium supplemented with 10% FBS. 24 h later, free DOX, DOX-loaded PEG-DiHyd-PLA, and PLA-PEG-PLA micelles were added into the wells at the same DOX concentration of 10 μg/mL. After being incubated at 37°C for 3 and 12 h, the cells were washed with PBS and fixed with PBS containing 4% formaldehyde for 15 min at room temperature. The cell nuclei were stained with 4′,6-diamidino-2-phenylindole (DAPI) for 15 min. The fluorescence signals of DOX and DAPI staining were investigated and imaged by the confocal laser scanning microscopy system (CLSM).

### Flow cytometry measurements

DOX-loaded micelles and free DOX uptake HepG-2 cells was assessed using the flow cytometry cell analyzer. HepG-2 cells were seeded in culture dish (5 × 10^5^ cells) in 1640 media and incubated for 24 h at 37°C. Then, HepG-2 Cells were treated with 1.5 mL of fresh cell culture medium and containing free DOX and DOX-loaded nanoparticles (equivalent concentration of DOX was 10 μg/mL) were added. After 48 h of incubation, the drug containing media was collected, and cells were trypsinized (without EDTA), centrifuged, washed with Binding Buffer (1X) for two times. Then collect the cells, and stained with Annexin V-FITC and propidium iodide (PI) for 20 min following the operating instructions (In the dark environment). Finally, the sample was tested by flow cytometry within 1 h.

### Statistical analysis

Origin 8.5 and GraphPad Prism 5.0 Software were used for the statistical analysis Differences were considered statistically significant at *P* < 0.05, via one-way ANOVA and Student's *t*-test.

## Results and discussion

### Synthesis of pH sensitive tri-block copolymers

**Figure 1:**
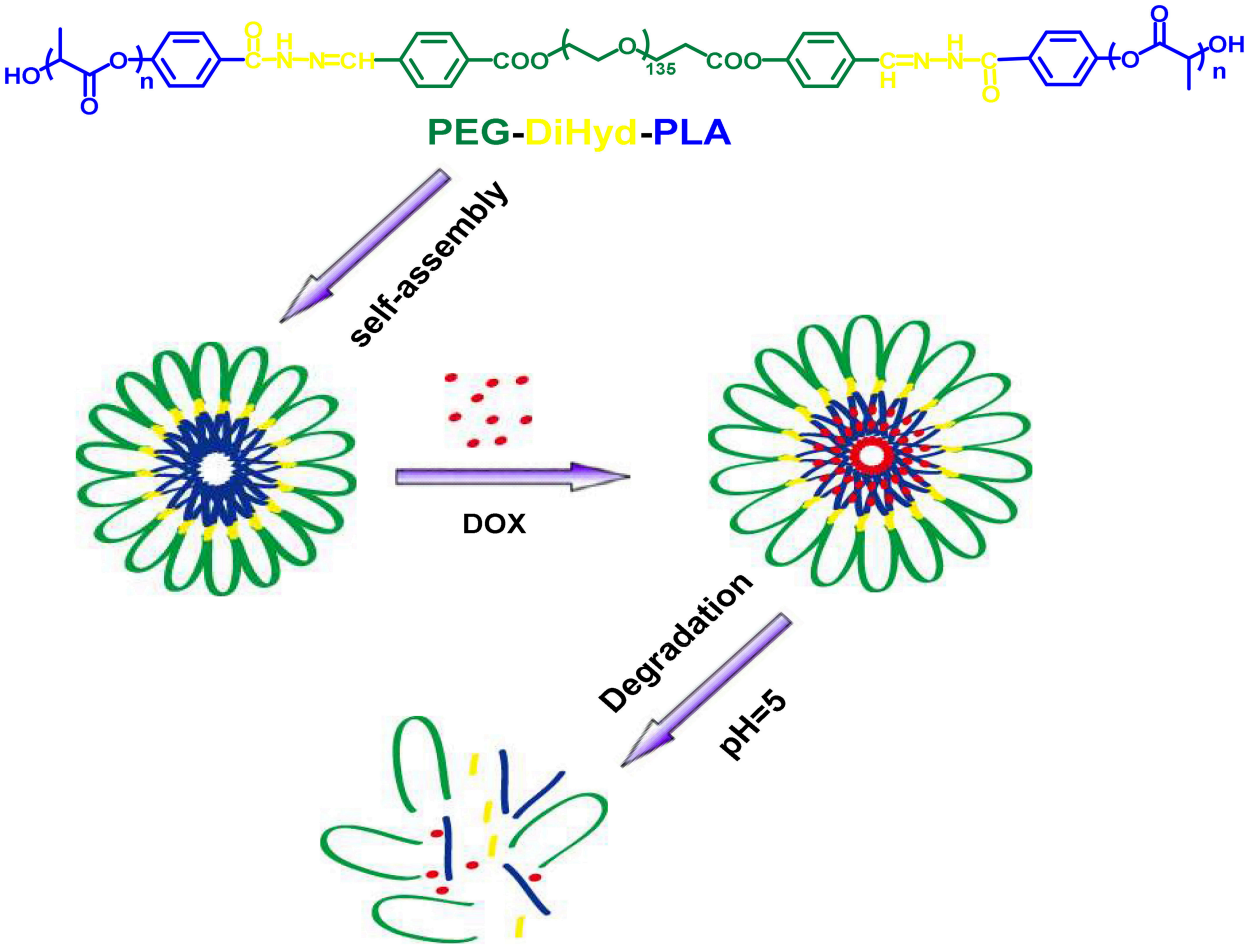
Schematic illustration of the formation of ABA triblock polymer micelle and its degradation in acidic condition.

**Figure 2:**
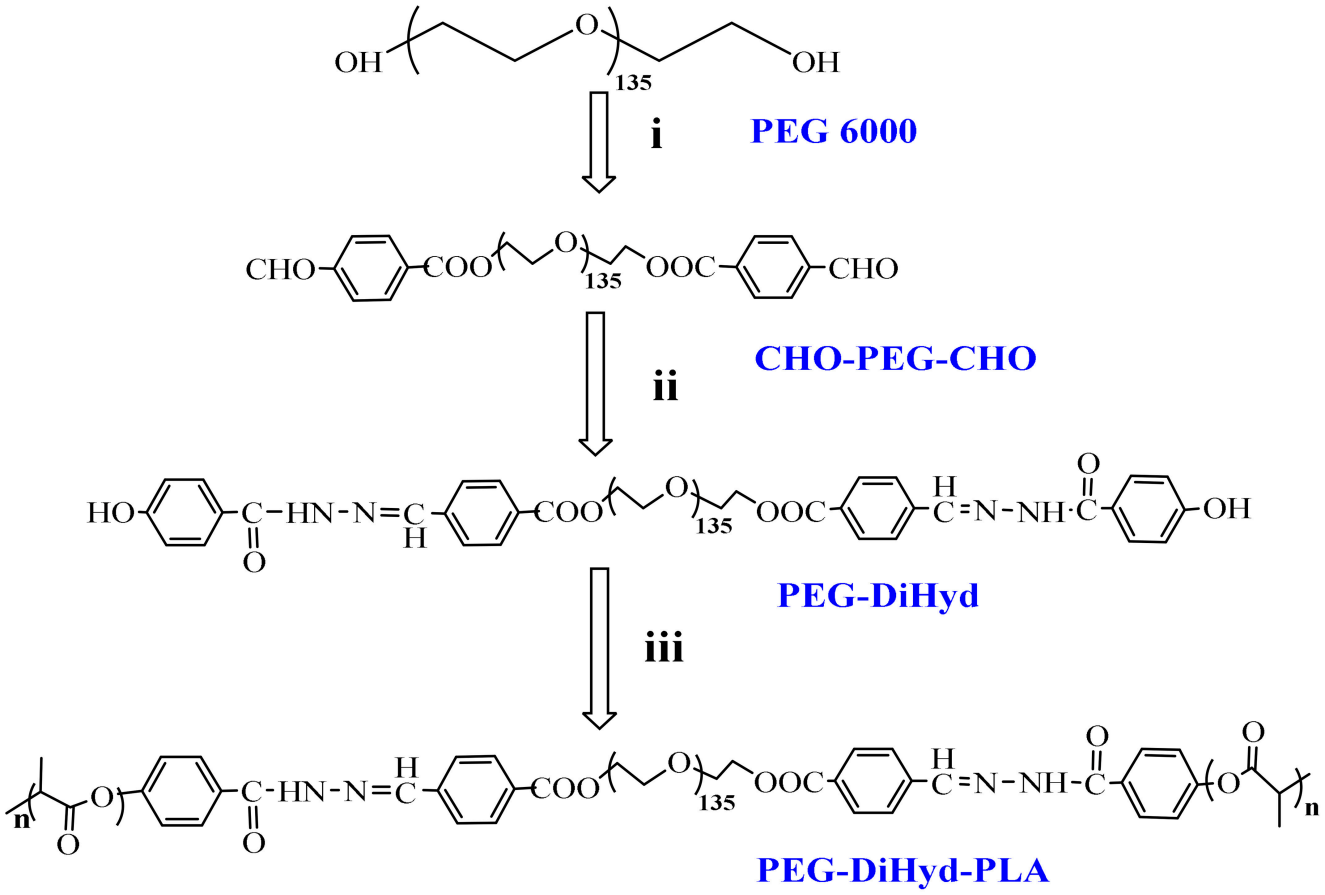
Synthesis route of copolymers PEG-DiHyd-PLA. **(i)** 4-Formylbenzoic acid, DCC, DMAP,R.T.; **(ii)** (4-Hydroxybenzoyl) hydrazine, Methanol, DMF, 68°C; **(iii)** Lactide, Sn(oct)_2_, Toluene,110°C, N_2_.

**Figure 3:**
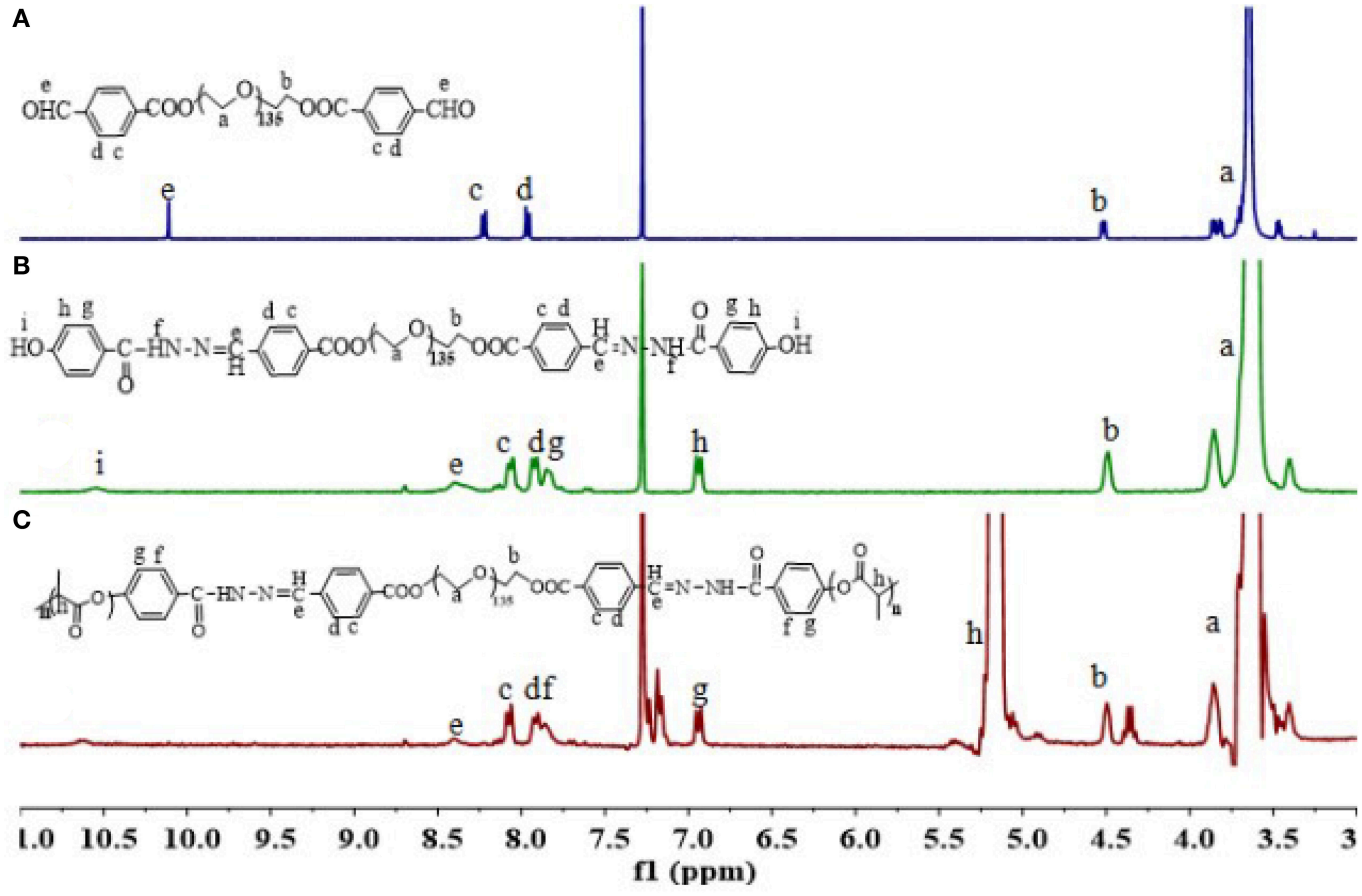
^1^H-NMR spectra of polymers CHO-PEG-CHO **(A)**, PEG-DiHyd **(B)**, and PEG-DiHyd-PLA **(C)**.

The triblock polymer with hydrazone on the backbone was prepared by a direct polymerization method (Figure 1). A hydrazone containing macro-initiator was synthesized first which followed by a ring opening polymerization. The synthesis is illustrated in Figure 2. CHO-PEG-CHO was firstly obtained by conjugation of PEG with 4-carboxybenzaldehyde. And 4-hydroxybenzoichydrazide was synthesized from methyl 4-hydroxybenzoate. The reaction between CHO-PEG-CHO and 4-hydroxybenzoichydrazide gave a molecule which embedded with hydrazone bond and terminal hydroxyl group. It could initiate ring-opening polymerization of lactide, to produce the copolymer PEG-DiHyd-PLA. The ^1^H-NMR spectra of CHO-PEG-CHO (Figure 3A) show signals characteristic of δ10.12 (Ar-CHO), δ8.21, δ7.97 (aromatic protons), δ3.65 (–OCH_2_CH_2_O–), and δ4.52 (–COOCCHO–). Through the above analysis, we can confirm the success of the synthesis of CHO-PEG-CHO. ^1^H-NMR showed that the macroinitiator PEG-DiHyd-Phenol was successfully synthesized, as indicated by the signal at δ8.41, which was assigned to hydrazone protons (Ar-CH = N) (Figure 3B). In the spectra of PEG-DiHyd-PLA (Figure 3C), characteristic signals of PEG (δ3.64), and poly (L-lactic acid) part (δ5.21, δ8.09, δ7.92, δ7.84, and δ6.98) appeared. The characteristic signals of hydrazone bond (δ8.42) were found to confirm the successful synthesis of PEG-DiHyd-PLA. PLA-PEG-PLA without pH-sensitive linkage was also synthesized.

Molecular weights of synthesized polymers are listed in Table 1. PEG-DiHyd-PLA-15K, PEG-DiHyd-PLA-18K, and PEG-DiHyd-PLA-20K are three copolymers with different molecule weights by designation, corresponding to the feed ratios 1:127, 1:169, and 1:197 between initiator and monomer, respectively. They have the same hydrophilic block PEG (Mn = 5,267 g/mol) and different hydrophobic blocks in different molecular weights. The molecular weights were calculated using the integral ratio between resonances at δ5.16 (one of methylidyne protons on PLA) and δ3.64 (methoxy proton of PEG) in ^1^H-NMR spectra. The calculated values based on ^1^H-NMR spectra were consistent with the designed ones. Gel permeation chromatography (GPC) measurements confirmed a unimodal distribution of Mn (16.3, 18.1, and 19.7 kg/mol, respectively) and narrow distribution [polydispersity index (PDI): 1.31, 1.71, and 1.44]. Therefore, well-defined biodegradable triblock polymers PEG-DiHyd-PLA was successfully synthesized.

**Table 1:** Synthesis of triblock polymers PEG-DiHyd-PLA.

**Polymer**	**(Mi/Mm)^a^**	**Yield (%)**	**Mn^b^ (kg/mol)**	**Mn^c^ (kg/mol)**	**PDI^d^**	**CMC^e^ (mg/L)**
PEG-DiHyd-PLA-15k	1:127	72	15.9	16.3	1.31	1.6
PEG-DiHyd-PLA-18k	1:169	82	19.3	18.1	1.71	0.87
PEG-DiHyd-PLA-20k	1:197	69	21.8	19.7	1.44	0.53

a *Feed ratios in mole between initiator and monomer*.

b *Calculated from ^1^H-NMR spectra*.

c *GPC results*.

d *is molecular weight distribution index*.

e *Critical micelle concentration determined using pyrene as a fluorescent probe*.

Micelles were formed by adding the amphiphilic polymers PEG-DiHyd-PLA into aqueous solution. The micelles have diameters ranged from 70 to 130 nm and increase with their molecular weight. The average particle size of the drug loaded micelles was larger than that of the blank micelles, which may be due to the larger volume of micelles after the hydrophobic core of the drug loaded with the micelles (Guo et al., 2012; Bao et al., 2014). The loading capacity of DOX loaded polymer PEG-DiHyd-PLA-18K micelles was about 4.3%, and the PLA-PEG-PLA was 2.7%. As shown in Table 1, the CMC of the polymeric micelles were 1.6, 0.87, and 0.53 mg/L for PEG-DiHyd-PLA-15K, PEG-DiHyd-PLA-18K, and PEG-DiHyd-PLA-20K, respectively, determined by fluorescence measurements using pyrene as a probe. CMC-values of the polymers decreased from PEG-DiHyd-PLA-15K to PEG-DiHyd-PLA-20K, which originated from the increased hydrophobic interaction of micelle core. Such a low value of CMC indicates an excellent stability under diluted conditions *in vivo*, which is so important for the micellar drug delivery system.

**Figure 4:**
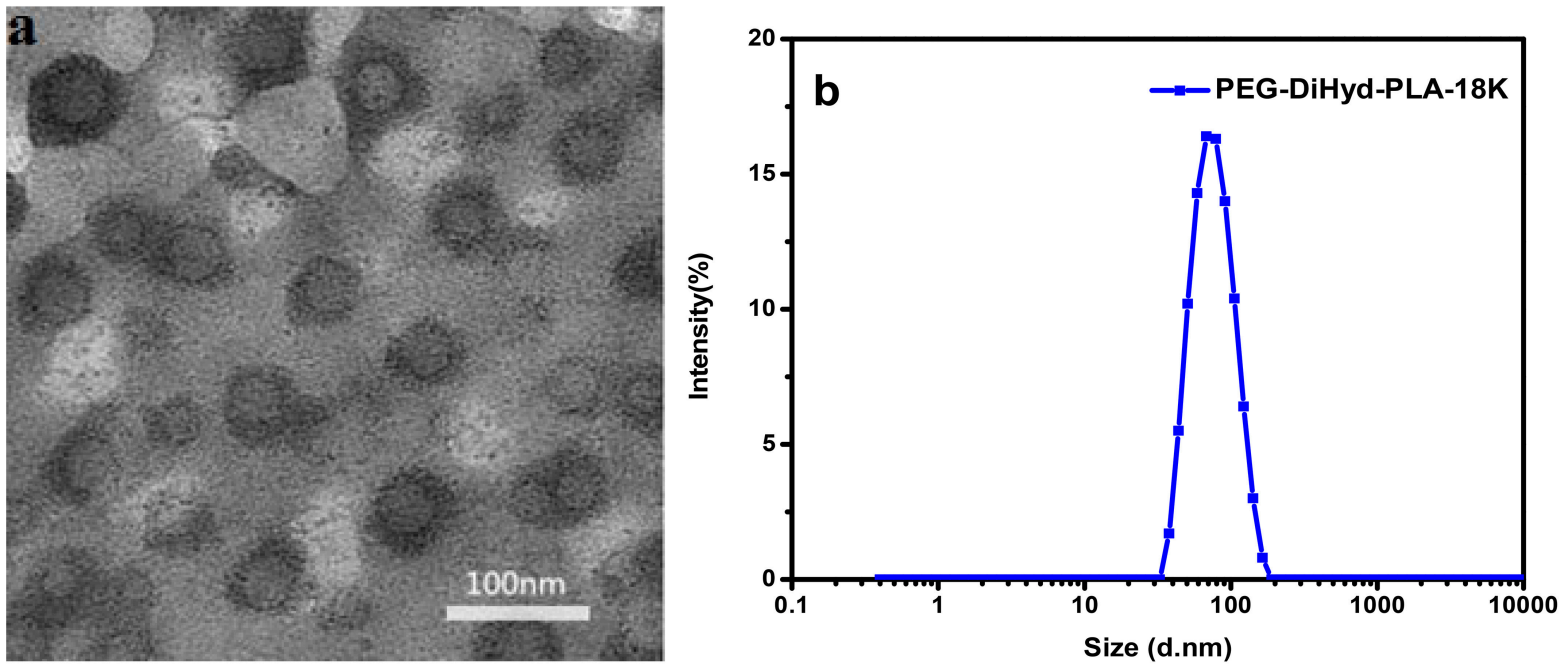
Images of micelles formed by PEG-DiHz-PLA-18K **(a)**. The particle size of PEG-DiHyd-PLA-18K micelles **(b)**.

The polymer PEG-DiHyd-PLA-18K micelles observed by TEM have a spherical core-shell like structure and are uniformly distributed (Figure 4a), the particle size of the polymer PEG-DiHyd-PLA-18K micelles measured by DLS is relatively small, both of which are about 70 nm, and the particle size distribution is narrow and PDI is lower than 0.2 (Figure 4b). It shows that the size of micelles is relatively uniform, and the particle size of micelle is basically consistent with the result of TEM determination. It was reported that nanoparticulate drug carriers can accumulate in tumor tissue via the enhanced permeability and retention (EPR) effect (Baish et al., 2011; Maeda and Matsumura, 2011) when their sizes are <200 nm. Thus, PEG-DiHyd-PLA micelles would effectively reach lesion sites, and achieve the goal of pH-controlled drug delivery.

### pH-triggered size change of the blank micelles

**Figure 5:**
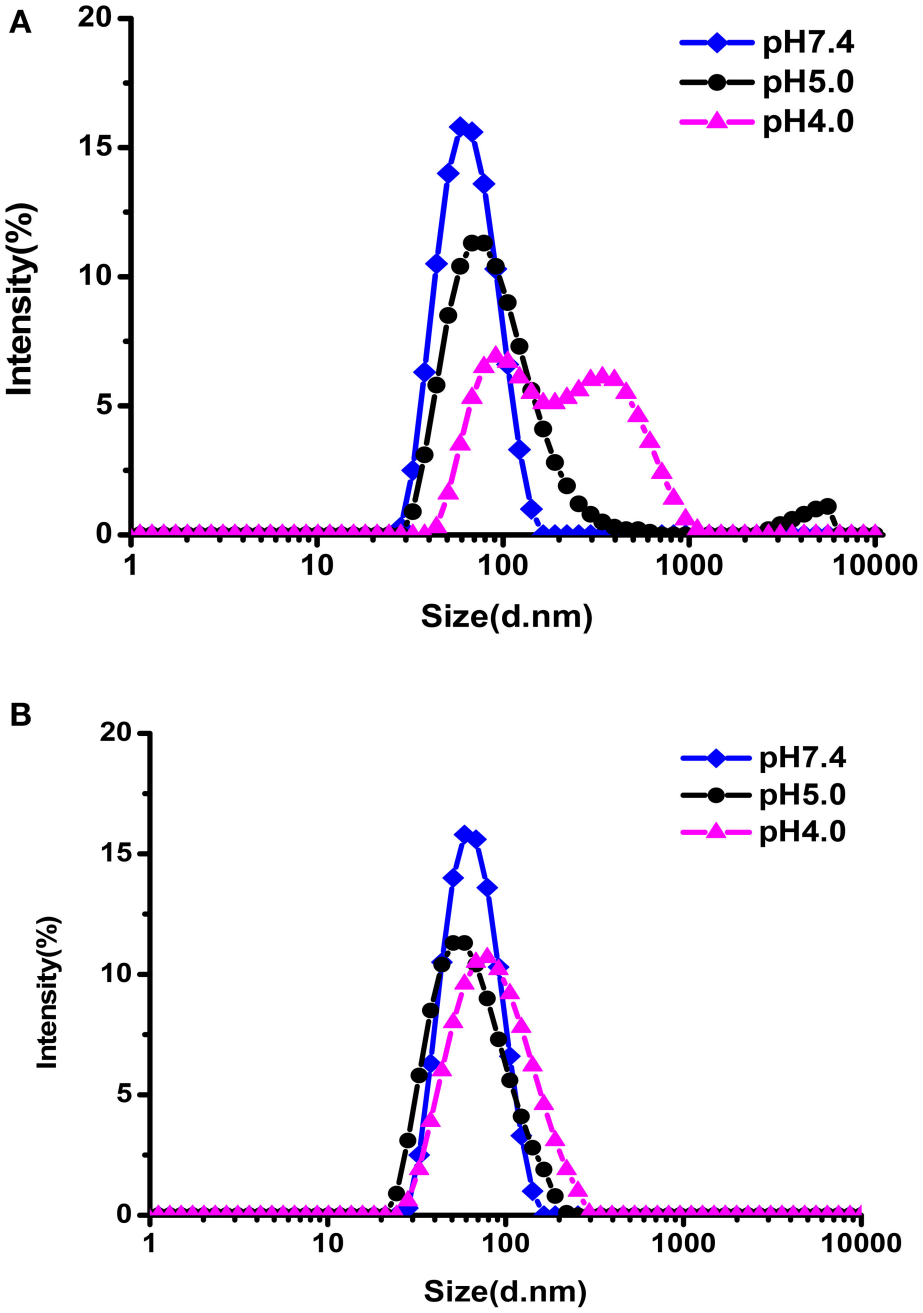
The size change of PEG-DiHyd-PLA-18K **(A)**; PLA-PEG-PLA-18K **(B)** micelles at different pH for 24 h.

The pH-responsive evolution of micelle was monitored by DLS measurements. As illustrated in Figure 5A, the size distribution of hydrazone-containing PEG-DiHyd-PLA-18K micelles underwent obvious changes under acidic conditions (pH 4.0, pH 5.0) while stable under physiological condition (pH 7.4). Multiple peaks appeared in the DLS curve and the solution became turbid due to precipitation, which was resulted from the decomposition of the pH-sensitive micelles in acidic environments. In contrast, PLA-PEG-PLA-18K micelles without hydrazone bonds kept almost unchanged under all pH conditions (Figure 5B) in 24 h. It is supported that the pH sensitive micelles will keep stable in blood circulation and protect their payload from being released before access targeting tumor tissue. After they encounter tumor tissue via EPR effector internalized by tumor cell where there is acidic condition, the loaded drug will be released instantly.

### *In vitro* controlled release of DOX

**Figure 6:**
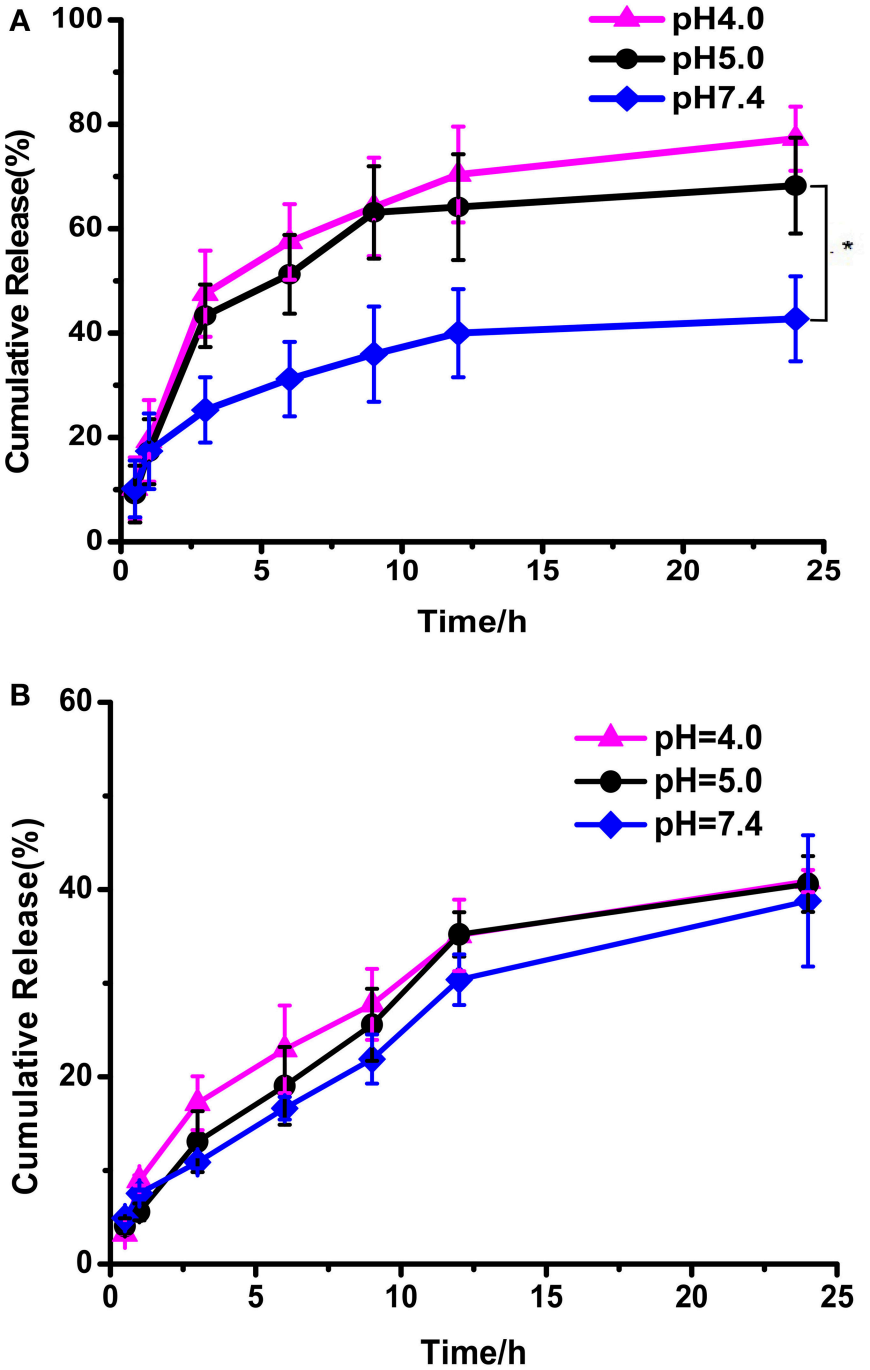
*In vitro* release of DOX from PEG-DiHZ-PLA-18K **(A)**; PLA-PEG-PLA-18K **(B)** micelles. Error bars indicate the standard error of the mean (SEM) for *n* = 3 independent experiments (^*^p < 0.05).

The *in vitro* drug release behaviors under various conditions were investigated (Figure 6A). DOX released from DOX loaded PEG-DiHyd-PLA-18K micelles at physiological pH was about ca. 38% in 24 h. The release rate was significantly promoted at pH 5.0 and 4.0, with accumulated release above 75% in 24 h, and there is no obvious difference between them. It is the pH-sensitive hydrazone bond results the pH-controlled drug release profile of polymeric PEG-DiHyd-PLA-18K micelles. Comparably, Figure 6B, the release of DOX from pH insensitive polymeric micelles showed a similar rate of release. Under different pH conditions, there was no pH-dependent release profile, with cumulative release of about 40% in 24 h. This results are consistent with the size changes in different conditions for the two types of micelles. The pH-responsive release of the hydrazone containing micelles might underwent a cleavage-disassociation-release process.

### MTT assay of DOX-loaded micelles

**Figure 7:**
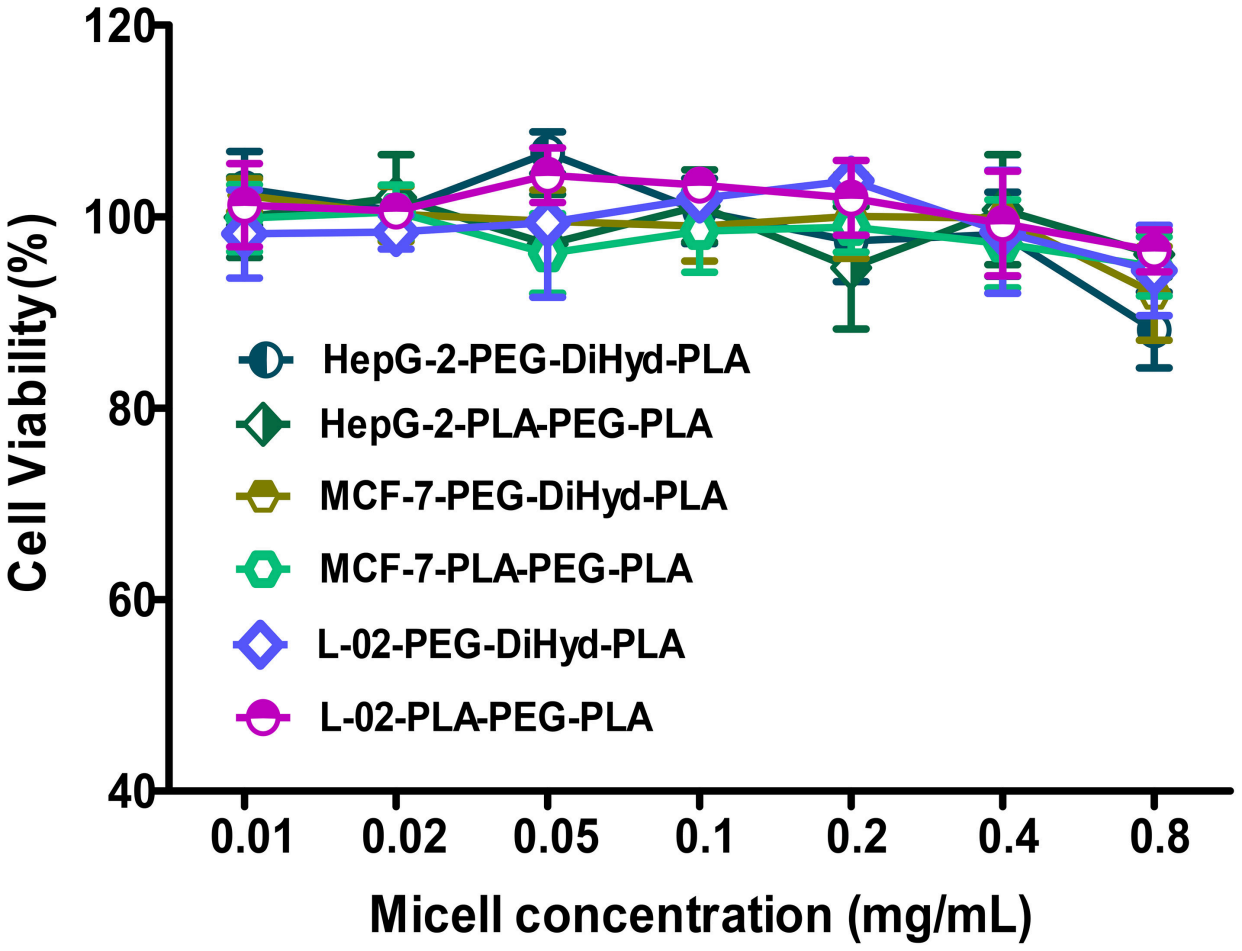
The cytotoxicity of blank PLA-PEG-PLA-18K, PEG-DiHyd-PLA-18K micelles incubated with HepG-2, MCF-7, and L-02 cells for 48 h. Error bars indicate the standard error of the mean (SEM) for (*n* = 3) independent experiments.

The cytotoxicity of the blank micelles were tested in MCF-7, HepG-2, and normal hepatocyte L-02 cells by a MTT assay. Cells viabilities of cells was above 90% for both blank PLA-PEG-PLA-18K and PEG-DiHyd-PLA-18K micelles following 48 h incubation (Figure 7), which meant that the blank micelles are remarkably no-toxic and biocompatible up to a concentration of 0.8 mg/mL.

**Figure 8:**
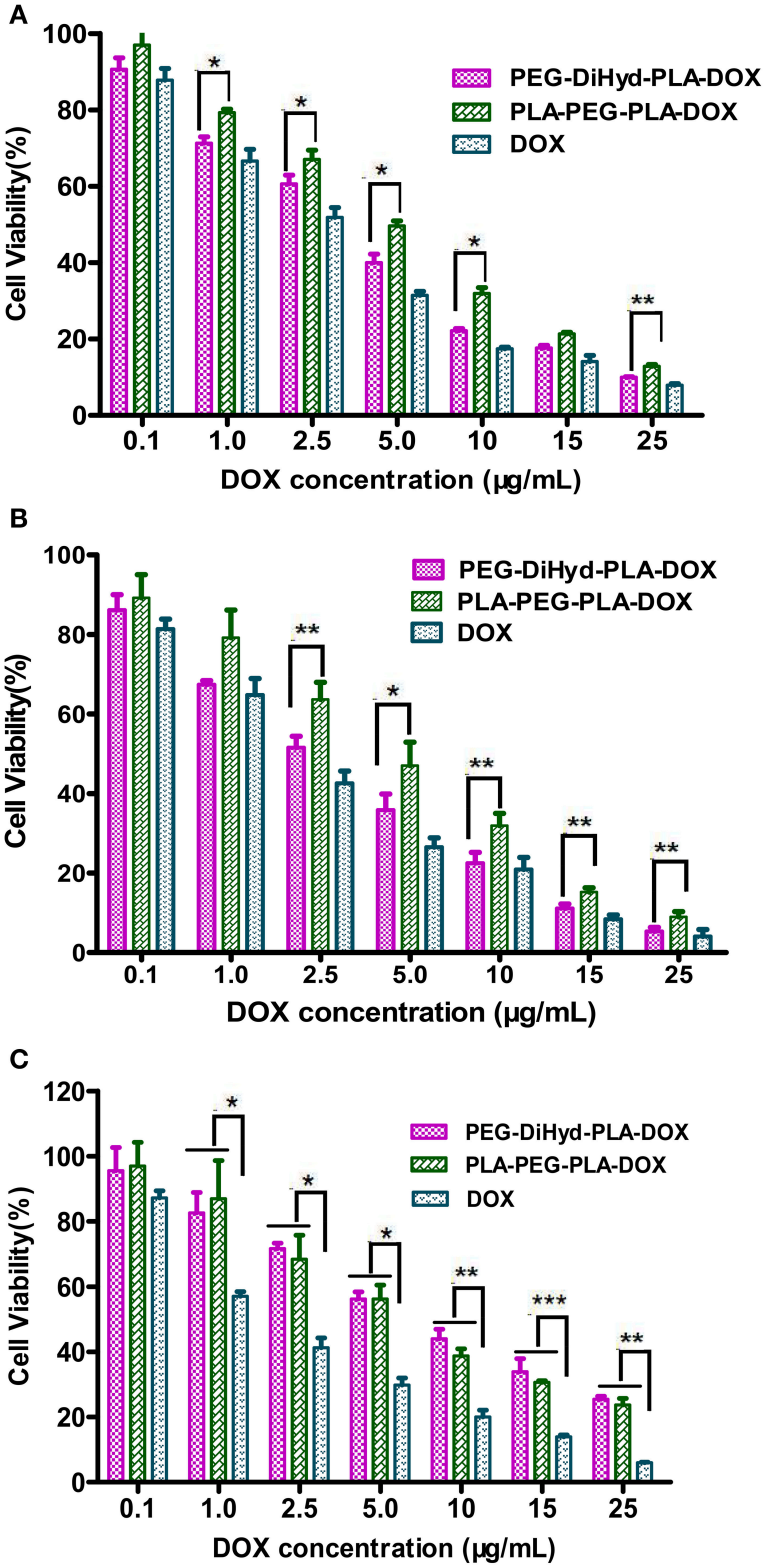
The cytotoxicity of DOX loaded PLA-PEG-PLA-18K, PEG-DiHyd-PLA-18K micelles and Free DOX incubated with HepG-2 cells **(A)**, MCF-7 cells **(B)**, and L-02 **(C)** at different DOX concentrations 48 h. Data were presented as mean ± standard deviation (*n* = 3) (^*^p < 0.05, ^**^p < 0.01, ^***^p < 0.001).

*In vitro* antitumor capability of PEG-DiHyd-PLA-18K based drug delivery systems are tested. Formulations including free DOX, DOX loaded PLA-PEG-PLA-18K, and PEG-DiHyd-PLA-18K micelles were evaluated against MCF-7, HepG-2, and L-02 cell lines. The IC_50_-values for various DOX for mutations and free DOX are summarized in Table 2, As shown in Figure 8, for all of the cells, the formulations showed a dose dependent cell proliferation inhibition behaviors after a 48 h incubation, free DOX showed higher *in vitro* toxicity to each cell, compared to the other two micelle formulations. DOX is a small molecule, so it can be quickly transported into cells and reach nuclei by passive diffusion (Cui et al., 2013). This is why the inhibition effect of free DOX was the strongest. While, for HepG-2 and MCF-7 cells (Figures 8A,B), pH sensitive DOX loaded PEG-DiHyd-PLA-18K micelles was more toxic than DOX loaded PLA-PEG-PLA-18K. Superior cell-killing capability of DOX loaded PEG-DiHyd-PLA-18K micelles may be due to the fact that entry of pH-sensitive micelles through endocytosis and drug release into the cytoplasm triggered by endosome pH are quick and efficient processes (Tang et al., 2011). As shown in Figure 8C, compared with free DOX, the DOX-loaded micelles exhibited significantly reduced cytotoxicity on L-02 cells, which is probably due to their slower uptake of DOX-loaded micelles by L-02 cells. While, there was no significant difference in the cytotoxicity between the two DOX-loaded micelles, which could be attributed to the limited of acid environment in L-02 cells compared with tumor cells (Qin et al., 2017).

**Table 2:** Half-inhibitory concentration (IC_50_) of loaded-DOX micelles and free DOX on HepG-2, MCF-7, and L-02 cells.

**IC_50_(μg/mL)**	**HepG-2**	**MCF-7**	**L-02**
	**48 h**	**48 h**	**48 h**
PLA-PEG-PLA-DOX	4.601	4.053	6.464
PEG-DiHyd-PLA-DOX	3.098	2.303	7.178
Free DOX	2.182	1.615	1.486

### Cellular uptake of the drug-loaded micelles

**Figure 9:**
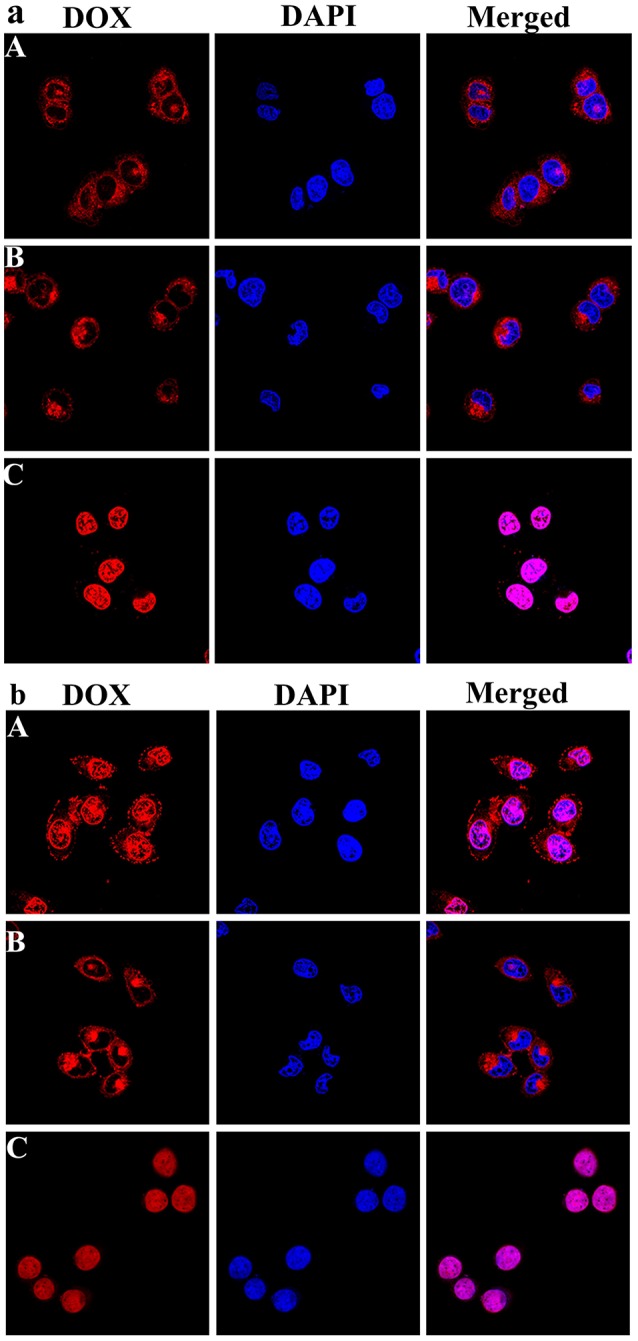
Representative CLSM images of HepG-2 cells incubated with DOX-loaded mPEG-DiHyd-PLA-18K Micelle solution **(A)**, DOX loaded PLA-PEG-PLA-18K micelle solution **(B)**, and free DOX **(C)** solution for 3 h **(a)** and 12 h **(b)**. The cellular nuclei and cytoskeleton of cells were stained with DAPI (blue) and DOX (red), respectively. DOX concentration is 10 μg/mL for any DOX containing solutions.

The intracellular localization and distribution of DOX-loaded micelles were investigated in HepG-2 cells using CLSM after incubation for 3 and 12 h (Figure 9). As shown in Figure 9a, for the DOX loaded micelles, most of the red fluorescence appears in the cytoplasm, and there is no obvious difference between the two drug loaded micelles. But with extended incubation time to 12 h, it was also observed that the pH sensitive DOX-loaded PEG-DiHyd-PLA-18K micelles showed relatively strong red fluorescence in the nucleus, while the red fluorescence of the DOX-loaded PLA-PEG-PLA-18K micelles was mainly in the cytoplasm and the nuclei were less (Figure 9b). That was due to the accelerated DOX release from hydrazone-containing micelles in the acidic tumor microenvironment. The accumulation of DOX-loaded micelles was lower than that of free DOX with the same incubation time, the possible reason is that free DOX transported into cells via a passive diffusion mechanism (Li et al., 2014). Compared to DOX-loaded PLA-PEG-PLA-18K micelles, the quick and efficient uptake of DOX from pH sensitive micelles greatly inhibited the growth of the tumor cell.

### Apoptosis of the drug-loaded micelles

**Figure 10:**
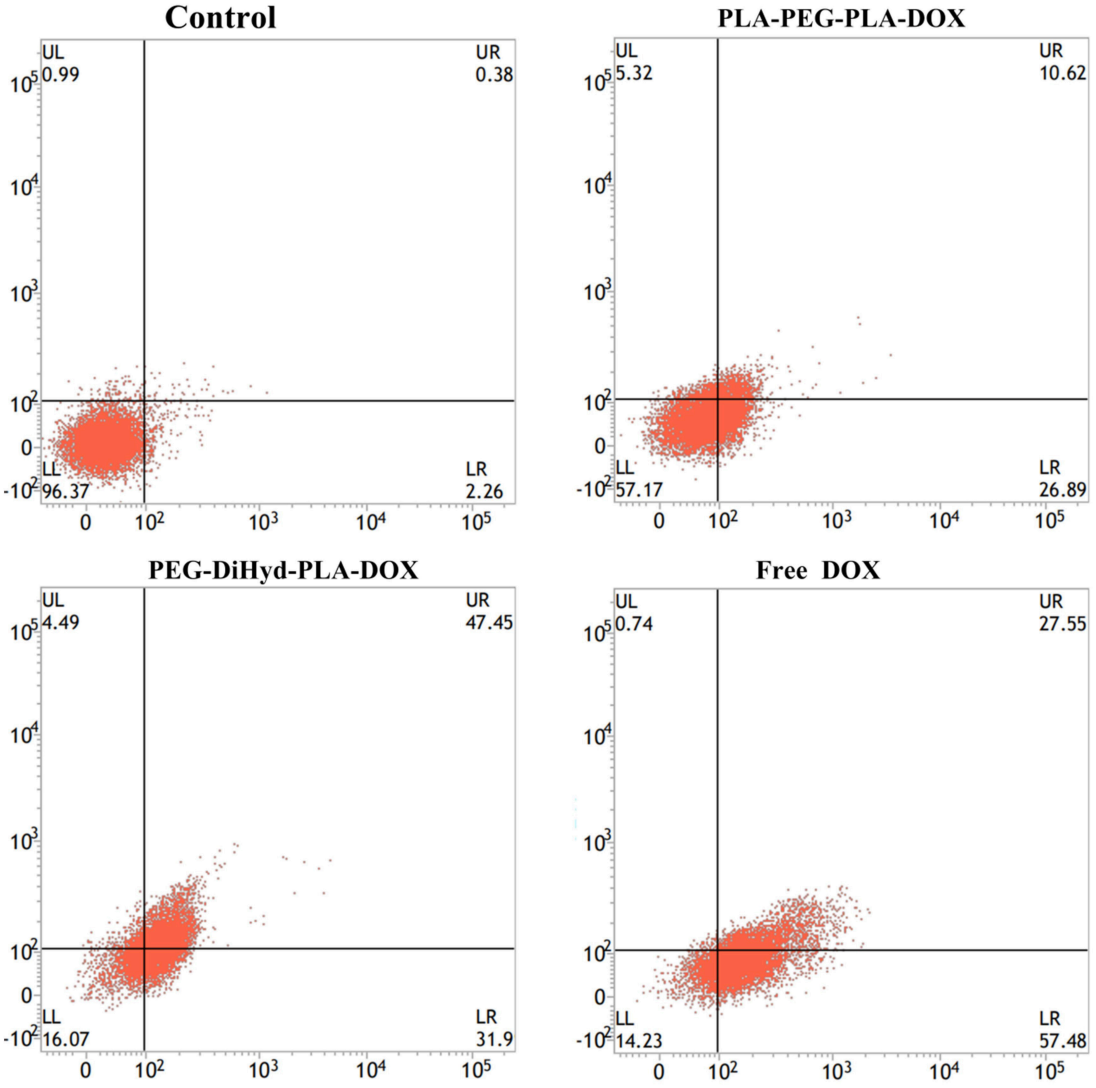
Apoptotic cell populations determined by flow cytometric analysis with Annexin V-FITC and PI staining after incubating HepG-2 cells for 48 h, DOX equivalent concentration was 10 μg/mL. The **lower-left** and **upper-left** quadrants in each panel indicate the populations of normal cells and necrotic cells, respectively. While the **lower-right** and **upper-right** quadrants in each panel indicate the populations of early and late apoptotic cells, respectively.

Apoptosis has been reported to be one of the primary mechanisms of action of DOX (Wang et al., 2017). Incubated with HepG-2 cells at a equivalent concentration of 10 μg/mL DOX for 48 h (Yang et al., 2016), the effect of free DOX, DOX loaded PLA-PEG-PLA-18K, and PEG-DiHyd-PLA-18K micelles on apoptosis was shown in Figure 10, the total apoptosis ratio of DOX-loaded PLA-PEG-PLA-18K micelles was about 38% (a sum of the early apoptosis ratio of 26.89% and the late apoptosis ratio of 10.62%). With the HepG-2 cells treated with pH sensitive DOX loaded PEG-DiHyd-PLA-18K micelles, there is a 78% apoptosis ratio which is higher than 38%, which is likely due to the e accelerated release of the drug molecules from micelles with acid-labile hydrazone linkage by sensing the acidic environment of the endosomal compartments. The higher apoptosis rate of free DOX with the same incubation time, most likely because free DOX could diffuse passively through cell membranes quicker, whereas drug loaded micelles were internalized into cells via slower endocytosis (Hu et al., 2010). Apoptosis experiment again confirms the superiority advantages of pH sensitive micelles in tumor targeting therapy.

## Conclusions

In this work, a type of pH-sensitive polymeric micelles was prepared as carrier of DOX. A triblock copolymer has one hydrophilic PEG segment and two hydrophobic PLA segments. pH-sensitive hydrazone bond was used to connect them together, denoted as PEG-DiHyd-PLA. The copolymercan self-assemble into micelles with uniformed size below 100 nm and narrow size distribution. The size of the hydrazone-containing micelles underwent obviously changes in mildy acidic environments while kept unchanged in the neutral. Almost no change was found for polymeric micelles without hydrazone (PLA-PEG-PLA). DOX was successfully loaded into the micelles and presented a more rapid and complete drug release in acidic condition (pH 5.0). The results of *in vitro* cell assay revealed that the blank micelles were non-toxic and good biocompatibility. DOX-loaded PEG-DiHyd-PLA micelles possessed higher anti-tumor activity to kill the MCF-7 and HepG-2 cells in comparison with DOX loaded PLA-PEG-PLA micelles and less cytotoxicity to normal L-02 cells at similar DOX concentrations. Confocal and apoptotic experiments also proved that superiority advantages of pH sensitive micelles for tumor therapy.

## Author contributions

PQ, performed the synthesis and characterizations; XW, performed the cell experiments; LL, designed the synthesis and wrote the paper; HY, performed the formulations; SS, designed the whole experiment.

### Conflict of interest statement

The authors declare that the research was conducted in the absence of any commercial or financial relationships that could be construed as a potential conflict of interest.
